# Positron Emission Tomography Imaging Reveals Auditory and Frontal Cortical Regions Involved with Speech Perception and Loudness Adaptation

**DOI:** 10.1371/journal.pone.0128743

**Published:** 2015-06-05

**Authors:** Georg Berding, Florian Wilke, Thilo Rode, Cathleen Haense, Gert Joseph, Geerd J. Meyer, Martin Mamach, Minoo Lenarz, Lilli Geworski, Frank M. Bengel, Thomas Lenarz, Hubert H. Lim

**Affiliations:** 1 Department of Nuclear Medicine, Hannover Medical School, Hannover, Germany; 2 Cluster of Excellence Hearing4all, Hannover Medical School, Hannover, Germany; 3 Department of Medical Physics and Radiation Protection, Hannover Medical School, Hannover, Germany; 4 Department of Otorhinolaryngology, Hannover Medical School, Hannover, Germany; 5 Department of Otolaryngology, Charité, University Medicine Berlin, Berlin, Germany; 6 Departments of Biomedical Engineering and Otolaryngology-Head & Neck Surgery, University of Minnesota, Minneapolis, Minnesota, United States of America; University of Manchester, UNITED KINGDOM

## Abstract

Considerable progress has been made in the treatment of hearing loss with auditory implants. However, there are still many implanted patients that experience hearing deficiencies, such as limited speech understanding or vanishing perception with continuous stimulation (i.e., abnormal loudness adaptation). The present study aims to identify specific patterns of cerebral cortex activity involved with such deficiencies. We performed O-15-water positron emission tomography (PET) in patients implanted with electrodes within the cochlea, brainstem, or midbrain to investigate the pattern of cortical activation in response to speech or continuous multi-tone stimuli directly inputted into the implant processor that then delivered electrical patterns through those electrodes. Statistical parametric mapping was performed on a single subject basis. Better speech understanding was correlated with a larger extent of bilateral auditory cortex activation. In contrast to speech, the continuous multi-tone stimulus elicited mainly unilateral auditory cortical activity in which greater loudness adaptation corresponded to weaker activation and even deactivation. Interestingly, greater loudness adaptation was correlated with stronger activity within the ventral prefrontal cortex, which could be up-regulated to suppress the irrelevant or aberrant signals into the auditory cortex. The ability to detect these specific cortical patterns and differences across patients and stimuli demonstrates the potential for using PET to diagnose auditory function or dysfunction in implant patients, which in turn could guide the development of appropriate stimulation strategies for improving hearing rehabilitation. Beyond hearing restoration, our study also reveals a potential role of the frontal cortex in suppressing irrelevant or aberrant activity within the auditory cortex, and thus may be relevant for understanding and treating tinnitus.

## Introduction

Starting from the 1980s, positron emission tomography (PET) has been used as a tool for imaging functional neuroanatomy [1]. Amongst others, studies of the auditory system have been conducted addressing basic neuroscience questions (e.g., attentional modulation of auditory activation) and clinical research [2]. However, functional magnetic resonance imaging (fMRI) has become the imaging modality of choice for many experimental questions, since it avoids radiation exposure. Nevertheless, PET retains considerable advantages for studies of the auditory system, as it is a quiet imaging technique and can be used for neural prosthetic patients for whom fMRI results in magnetic interference and safety issues with the implanted device [2].

PET and single-photon emission computed tomography (SPECT) are alternative imaging approaches that have already been safely and successfully used to image brain function in response to auditory implant stimulation. This imaging capability could have a significant clinical impact considering that over 300,000 patients have been implanted with auditory implants (i.e., at the cochlear, brainstem, and midbrain levels [3]). In particular, PET and SPECT could have the potential to characterize and even diagnose the functional effects of artificial stimulation of the brain, which in turn can guide the development of better stimulation strategies and identification of more appropriate brain targets for these implants.

In terms of patients implanted with the cochlear implant (CI), which consists of an electrode array positioned into the cochlea that electrically activates nearby auditory nerve fibers, PET and SPECT have begun to reveal differences in activation patterns between good and poor performing patients. Distinct patterns of regional brain activity have been described, for example, in the context of (i) cross-modal plasticity during rehabilitation [4–6], (ii) improved results after implantation of pre- versus post-lingually deaf patients [7–9], and (iii) differences between monaural and binaural stimulation [10]. One consistent finding across studies is that patients with higher speech perception exhibit greater bilateral activation across the auditory cortex and what has been classified as the temporal voice area (TVA), which spans Brodmann Area (BA) 21, 22, 41, and 42 [4, 6–8, 11, 12]. There have also been a few PET or SPECT imaging studies of patients with the auditory brainstem implant (ABI), which consists of an electrode array positioned on the surface of the brainstem to electrically activate neurons within the cochlear nucleus and has produced activation of auditory temporal cortices [13–17]. However, in one study it was shown that there are no differences in activation between voice and non-voice stimulation with the ABI as well as a lack of activation of some TVA areas typically observed in normal hearing subjects, which could relate to the limited speech perception achieved by most ABI patients [13]. More recently, a few deaf patients have been implanted with the auditory midbrain implant (AMI), which consists of an electrode array implanted into the inferior colliculus [18–20]. PET imaging can now be performed in these AMI patients.

We had a unique opportunity to expand upon these previous PET imaging studies. We identified a group of auditory implant patients with varying amounts of loudness adaptation in response to continuous stimulation (i.e., the perceived sound decreases or disappears as the same stimulus is presented over time; [21]). These patients provided a way to identify brain regions involved with the suppression or loss of sound perception. A few previous studies have shown that lesions of the frontal cortex leads to increased auditory cortical activity to sound stimuli, suggesting the inhibitory or gating role of the frontal cortex on ascending auditory information [22, 23]. However, we could directly assess in these implant patients if frontal cortex activity is increased while auditory cortex activity is decreased during loudness adaptation or suppression. Furthermore, all of these patients observed no loudness adaptation to speech input, which stimulated the same set of electrode sites in each patient but just with a different temporal and spatial pattern of electrical pulses (i.e., multi-tone stimulus versus speech inputted into the implant processor produces a different pulse pattern on the same electrode sites). Thus, we were able to show how drastically different cortical regions are activated by simply adjusting the temporal and spatial pattern of pulses presented to a given brain location, revealing the critical importance of identifying appropriate implant stimulation strategies to achieve effective activation of the auditory system.

Another unique aspect of our study is that we selected a group of deaf patients who are implanted with electrode arrays in different brain locations (i.e., CI, ABI, and AMI patients). This imaging study provides the opportunity to assess cortical activation effects across different brain stimulation locations and is the first to image the effects of AMI stimulation. Although we only had five patients, we used a specific PET imaging protocol to enable sufficient statistical evaluation on a patient-by-patient basis, and thus we could compare brain activation effects between each patient. Even across three different implant types, we observed that greater bilateral activation of the auditory cortex and TVA areas correlated with better speech perception, consistent with what has been shown for CI patients.

## Material and Methods

The study has been approved by the local ethics committee of Hannover Medical School and by the German Federal Office for Radiation Protection (reference number: Z5-22463/2-2008-015). Before participation in the study, all patients gave informed written consent.

### Patients

Five postlingually deafened patients (2 males, 3 females) with a mean age of 55 years (range 30–76) were included. One patient was implanted with a CI, two patients were implanted with an ABI, and two patients were implanted with an AMI (see Table 1 for patient number and side of implantation). Patient 1 had inner ear hearing loss with an unknown cause. In patient 2, deafness occurred after meningitis and middle ear inflammation necessitating bilateral mastoidectomy. Patients 3, 4, and 5 suffered from neurofibromatosis type II and were implanted with their device after removal of bilateral acoustic neuromas. Auditory implantation had been performed in the mean 5.1 years (SD 2.7, range 1.8–8.9) before PET scanning. Patients 2, 3, and 5 perceive a tinnitus. Speech perception and loudness adaptation were assessed in all patients one day before the PET investigation and checked for consistency throughout the PET investigation as further described below.

**Table 1:** Patient characteristics and activation in auditory cortex during stimulation with speech.

Patient no., handedness, auditory implant, speech score	Activation in PET ^a^									
	Side	x ^b^	y ^b^	z ^b^	Z value	T value	BA41 ^c^	BA42 ^c^	BA22 ^c^	BA21 ^c^
No. 1, ambidexter, CI left, 82%	left	-50	6	-10	5.74	19.95	76	336	1241	1592
	right	70	-28	4	5.67	18.97	0	0	812	1072
No. 2, right handed, ABI 1 left, 58%	left	-70	-24	6	4.23	15.09	0	89	382	300
	right	58	-32	8	4.36	16.91	33	211	589	460
No. 3, right handed, ABI 2 left, 55%	left	-58	-32	8	5.09	11.73	107	206	1075	989
	right	64	-2	-6	4.70	9.45	0	40	719	541
No. 4, right handed, AMI 1 left, 5%	left	-66	-24	8	4.11	6.96	0	43	568	97
	right	74	-18	2	4.59	8.91	0	24	549	300
No. 5, right handed, AMI 2 left, 0%	left						0	0	0	0
	right						0	0	0	0

^a^ Threshold for statistical inferences: p<0.001.

^b^ MNI coordinates.

^c^ Voxels in Brodmann Areas.

### Speech performance

For assessing speech perception performance, a list of number-word pairs was presented via the implant at a comfortable loudness level. This level was determined by having the patient point to a value of 4 on a loudness scale (0: inaudible, 1: very soft, 2: soft, 3: medium, 4: comfortably loud, 5: loud, 6: very loud; includes 0.5 steps). A value of 4 corresponds to what the patient is comfortably hearing on a daily basis. There were no lip-reading cues since the recorded speech sounds were presented from an audio device into the implant processor via a direct cable input.

The speech list consisted of numbers between 13 and 99 and simple words to create a number-word pair (e.g., 50 bottles). The speech list was developed by a speech therapist at Hannover Medical School who has had more than 10 years of experience working with CI, ABI, and AMI patients. Hearing performance assessed with this list revealed wide differences in speech perception abilities across our implanted patients that we could correlate with differences in PET imaging patterns. Each number-word pair out of 100 different pairs was presented to the patient and the patient had to verbally repeat what was heard. The speech score was calculated as a percentage of correctly identified number-word pairs out of 100 different pairs. Speech scores are listed in Table 1.

### Loudness adaptation

**Fig 1:**
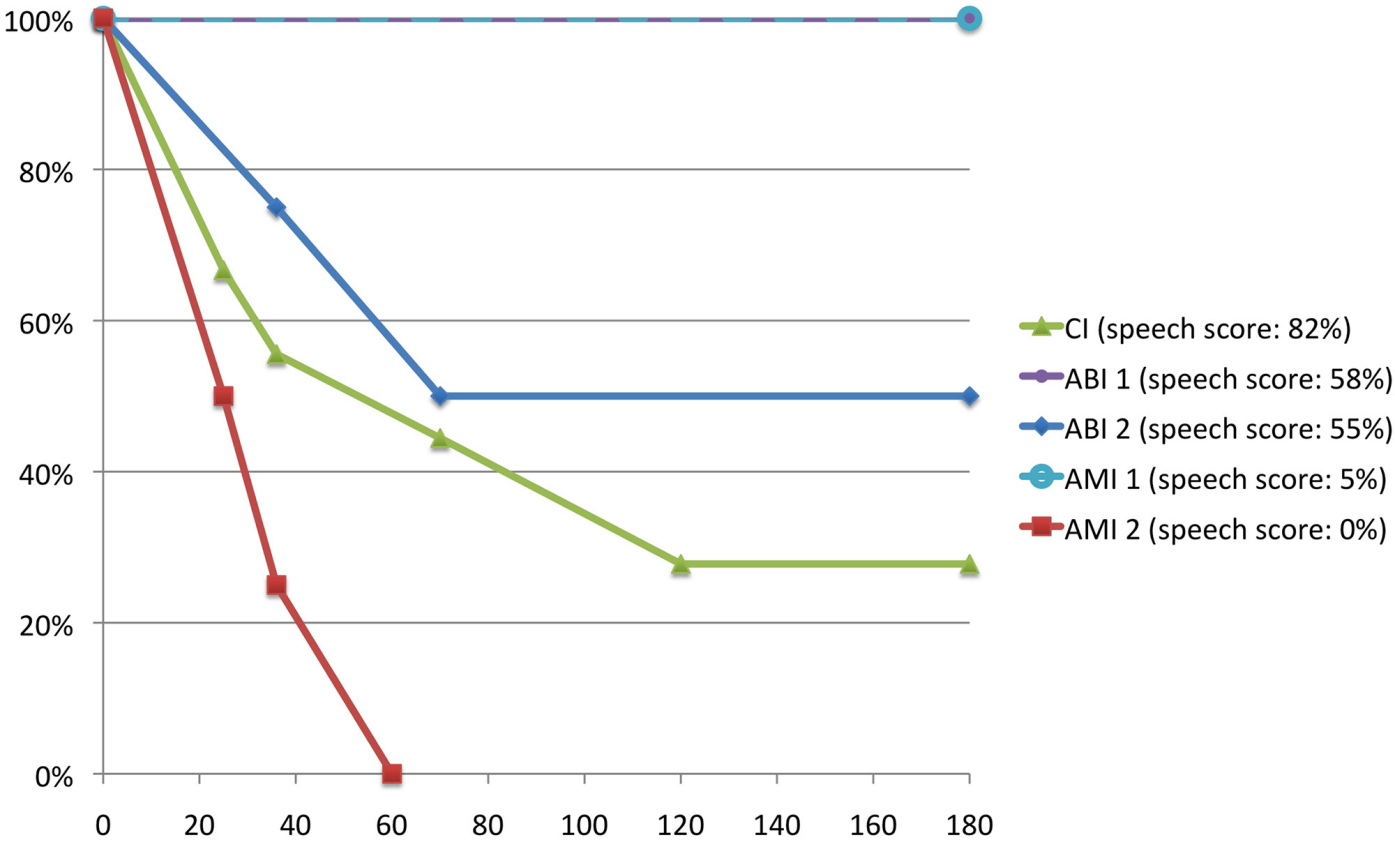
Time course of loudness adaptation in auditory implant users. Ordinate displays the percentage relative to comfortable loudness that is maintained over time (in seconds along the abscissa) in response to continuous multi-tone complex stimulation. Speech perception scores for each implant user taken from Table 1 are listed in the legend to the right of the figure.

The extent of loudness adaptation was assessed psychophysically by presenting a continuous sound and having the patient rate the loudness over time. The sound was a continuous multi-tone complex (MTC) with frequencies at 300, 1200, 2500, and 5000 Hz. These frequencies were selected because they caused varying extents of adaptation across all five of our patients and spanned across the frequency range used for the different stimulation sites in each patient. The same loudness scale as used in the evaluation of speech performance was applied for assessing loudness adaptation. The loudness level of the MTC was adjusted until the patient pointed to a level of 4 corresponding to what the patient is comfortable hearing on a daily basis. Then after a break of a few minutes to allow for recovery, the sound was presented at that comfortable level and loudness ratings were obtained over 180 seconds. The loudness value over time for each patient was measured two times, averaged, and plotted on a normalized scale from 0 to 100%, as shown in Fig 1. Table 2 lists the extent of adaptation as complete, partial, or none based on the curves shown in Fig 1. Only four patients are listed in Tables 2 and 3 since one patient did not perform PET imaging for the adaptation part.

**Table 2:** Activation or *de*activation in auditory cortex during stimulation with multi-tone complex.

Patient no., auditory implant, adaptation	Activation in PET											
	Side	*De-/*activation	x ^a^	y ^a^	z ^a^	Z value	T value	p-level ^b^	BA41 ^c^	BA42 ^c^	BA22 ^c^	BA21 ^c^
No. 1, CI left side, partial	left							0.05	0	0	0	0
	right	Activation	34	16	60	4.27	7.56	0.05	0	0	522	0
No. 2, ABI 1 left side, none	left							0.001	0	0	0	0
	right	Activation	62	-32	8	4.33	9.28	0.001	120	38	93	83
No. 3, ABI 2 left side, partial	left	Activation	-62	-46	-24	2.72	3.43	0.05	0	0	0	471
	right	Activation	32	70	0	3.76	5.82	0.05	329	0	867	1584
	right	Activation	62	-46	30	2.30	2.73	0.05	0	27	0	0
No. 5, AMI 2 left side, complete	left	*Dea*ctivation	-50	-44	14	4.40	7.42	0.001	100	179	190	617
	right							0.001	0	0	0	0

^a^ MNI coordinates.

^b^ Note that different thresholds (p levels) for statistical inferences were used across patients.

^c^ Voxels in Brodmann Areas.

**Table 3:** Activation in frontal cortex (BA 9, 10) during stimulation with multi-tone complex.

Patient no., auditory implant, adaptation	Activation in PET ^a^							
	Side	x ^b^	y ^b^	z ^b^	Z value	T value	BA9 ^c^	BA10 ^c^
No. 1, CI left side, partial	left						0	0
	right	34	16	60	4.27	7.56	198	0
No. 2, ABI 1 left side, none	left						0	0
	right						0	0
No. 3, ABI 2 left side, partial	left						0	0
	right	34	68	0	3.75	5.79	0	16
No. 5, AMI 2 left side, complete	left	-40	42	40	3.27	5.00	1	0
	left	-12	52	6	3.56	5.87	0	217
	right	28	58	16	3.51	5.71	0	104

^a^ Threshold for statistical inferences: p<0.001.

^b^ MNI coordinates.

^c^ Voxels in Brodmann Areas.

### PET scanning

For data acquisition, a Biograph LSO Duo PET/CT (Siemens, Erlangen, Germany) was used. Each patient was scanned during three conditions with six repetitions for each condition [24, 25]: (A) silence, (B) stimulation with MTC, and (C) stimulation with speech (i.e., number-word pairs). PET scans were performed in two sessions separated by an approximately two-hour break on the same day with the sequence: ABBABBABB and ACCACCACC. Before each session, a low-dose CT was acquired for later attenuation correction. O-15-oxygen was produced at an on-site cyclotron and was transformed to O-15-water at the bedside of the PET scanner [26]. For each PET scan, 740 MBq O-15-water was automatically injected intravenously as a bolus within 7 sec.

Fifteen seconds after the start of the O-15-water injection, which accommodates the circulation time before the tracer appears in the brain, a 90-sec 3-D list mode acquisition was begun. Acoustic stimulation with either the MTC or speech list that was initiated 60 sec before the start of the tracer injection and lasted until the end of the scan, totalling a duration of 165 sec. We presented the MTC 75 sec before the start of the PET acquisition to ensure that sufficient adaptation had already occurred when imaging the brain. For consistency, we also performed the same timing protocol for the speech scans. Six different speech lists consisting of randomly selected and different number-word pairs from the 100 pairs used in the speech performance test were presented for the six speech scans. The same six lists were used for all patients. All acoustic stimuli were presented at a comfortable loudness (i.e., a loudness level of 4 as used in the testing sessions for speech performance and loudness adaptation) that was checked with the patients before and after the scanning sessions.

To ensure the patient was awake and attending to the acoustic stimulus, after the end of each scan we had the patient indicate as to what was heard within the last three minutes. We used signs to instruct the patients in how to respond and practiced with the patients several times before the scanning sessions. For the MTC scans, the patient displayed an open hand if no sound was heard and one finger if a sound was heard. For the speech scans, the patient displayed an open hand for no sound, one finger for a sound that was heard but not understood, two fingers for a sound in which parts of it were understood, and three fingers if the sound was well understood. Additionally, a webcam was used to monitor the digital control panel of the patient's speech processor to ensure that the implant was delivering stimulation during the appropriate periods. Between tracer applications, an interval of at least 10 minutes was provided to the patients to allow for sufficient decay of tracer radioactivity and recovery from acoustic stimulation.

### Data analysis

PET data were reconstructed using an OSEM 2-D algorithm (3 iterations, 8 subsets), including CT based attenuation correction.

Data analysis was performed using statistical parametric mapping software (SPM2, Wellcome Trust Center for Neuroimaging, London, UK). All scans were spatially normalized to the anatomical stereotaxic standard space according to the Montreal Neurological Institute employing the cerebral blood flow PET template of SPM2. Thereafter, scans were smoothed using an isotropic Gaussian filter kernel with a full width at half maximum of 20 mm. After proportional scaling of scans, single subject analyses (unpaired t-test) were performed for statistical comparison of conditions. Statistical inferences were based on a threshold of p<0.001 uncorrected for multiple comparisons (Z>3.09) on voxel level [27, 28]. Activation as well as deactivation during the respective stimulation conditions were defined compared to the silence condition.

Automated anatomical labelling was used for spatial assignment of significant effects [29]. A volume of interest (VOI) template reflecting different BAs was employed for that purpose. The number of voxels within a relevant VOI (e.g. BA 41 reflecting primary auditory cortex) was extracted as the extent of activation (i.e., cluster size). Cluster size of different brain regions were correlated with our values for speech score and loudness adaptation using JMP10 software (SAS Institute Inc.). Based on findings from previous studies described in the Introduction and the consistent effects observed in our data, we analysed and present these correlations for specific temporal (BA 21, 22, 41, and 42) and frontal (BA 9 and 10) cortices.

## Results

### Hearing performance with auditory implant

The five patients had varying levels of speech scores and loudness adaptation (Fig 1). The patients are listed in the legend of Fig 1 and in Tables 1–3 in order of high to low speech scores. Patients 2 and 4 (ABI 1 and AMI 1, respectively) had no adaptation. Patients 3 and 1 (ABI 2 and CI, respectively) had partial adaptation (down to 50% and 30%, respectively). Patient 5 (AMI 2) had complete adaptation. Patient 1 is one of the top performing CI patients in our clinic but had one of the largest amounts of loudness adaptation; thus, extent of loudness adaptation to a continuous multi-tone stimulus does not necessarily predict hearing performance to speech stimuli. On the other hand, Patient 5 had complete loudness adaptation and the worst speech score. Further studies are needed to determine if complete adaptation correlates with poor hearing performance across patients and implant types. The average speech score was 40% (SD: 36, range: 0–82%).

### Correlation between speech performance and auditory cortical activation

All four patients with at least minimal speech understanding (i.e., score ≥5%) showed bilateral activation of BA 22 and 21 together with unilateral or bilateral activation of BA 42 during speech stimulation. It was more difficult to detect activation in BA 41 due to its smaller size but typically there was at least unilateral activation across patients, except for Patient 4. There was no significant activation of any of these brain regions in Patient 5 who had a speech score of 0%. See Table 1 for further details.

**Fig 2:**
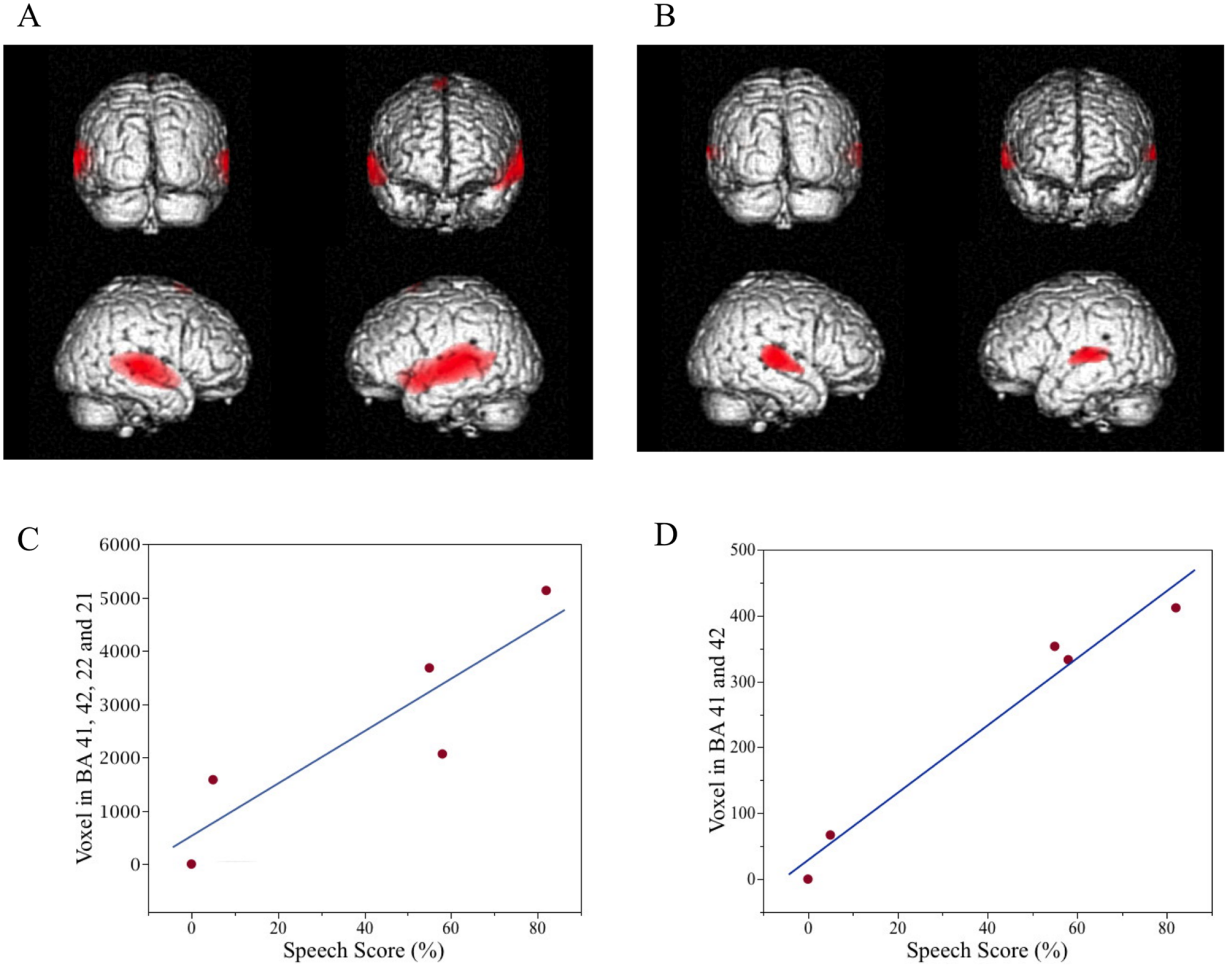
Correlation between speech understanding and extent of auditory cortex activation during speech stimulation. A CI user with good speech understanding (82%) exhibited large bilateral auditory cortex activation (Fig 2A), whereas an AMI user with poor speech understanding (5%) exhibited relatively small activated areas (Fig 2B). For the entire group, a significant correlation (R^2^ = 0.80, p = 0.042) between speech understanding (speech score) and extent of auditory cortex activation was observed for voxels within BA 41, 42, 22 and 21 (i.e., temporal voice area) (Fig 2C). A stronger significant correlation (R^2^ = 0.97, p = 0.002) was observed when plotting voxels only within BA 41 and 42 (Fig 2D).

The largest area of significant activation across BA 41, 42, 21, and 22 occurred for Patient 1 who had the highest speech score (Fig 2A), while the smallest measurable area was observed for patient 4 who had the lowest speech score (Fig 2B). Patient 5 had no measurable auditory cortex activity. Linear regression analysis revealed a significant correlation (R^2^ = 0.80, p = 0.042) between speech score and the number of activated voxels across BA 41, 42, 22, and 21 (Fig 2C). A stronger correlation (R^2^ = 0.97, p = 0.002) was observed between speech score and the number of activated voxels across only BA 41 and 42 (Fig 2D).

### Correlation between loudness adaptation and auditory or frontal cortical activation

**Fig 3:**
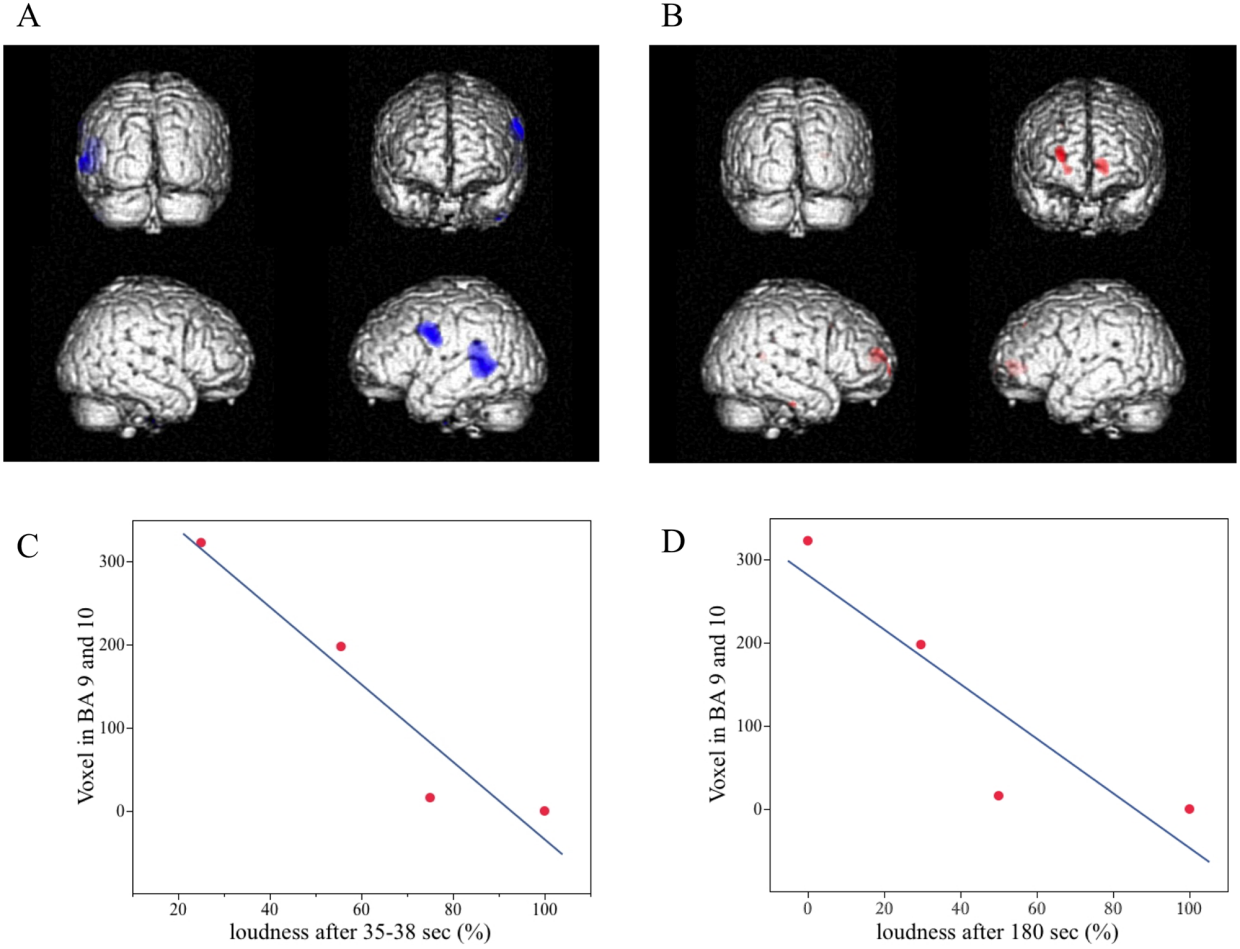
Correlations between loudness adaptation and cortical activation during multi-tone complex stimulation. A deactivation of the auditory cortex was seen in a patient with complete loudness adaptation (Fig 3A). In this patient, a significant (p<0.001) activation of the ventral frontal cortex was observed (Fig 3B). For the entire group, a significant negative correlation (R^2^ = 0.91, p = 0.045) between loudness maintenance at 35–38 sec presentation of the multi-tone complex (abscissa; taken from Fig 1) and the extent of frontal cortex activation (ordinate; voxels in BA 9 and 10) was observed (Fig 3C). For loudness maintenance at 180 sec, a similar but non-significant trend (R^2^ = 0.80, p = 0.106) was also observed (Fig 3D)

All three patients with loudness adaptation showed no supra-threshold activation (i.e., p>0.001, Z<3.09) of BA 41, 42, 22, and 21 during MTC stimulation. However, two of these patients with partial loudness adaption (30 or 50%) showed temporal cortical activation at a lower level of significance (p<0.05, Z>1.66), and the one patient with complete adaptation (0%) showed deactivation of the temporal cortex at a level of p<0.001, in which the latter is shown in Fig 3A. The one patient without loudness adaptation showed a significant activation (p<0.001) across BA 41, 42, 22, and 21. Table 2 provides the cluster size of activation/deactivation (i.e., number of voxels) in BA 41, 42, 22, and 21 at the respective significance levels.

In patients with loudness adaptation, activation of the frontal cortex (BA 9 and 10; see Table 3) was observed during MTC stimulation, in which the greatest activation was observed in the patient with complete loudness adaptation (Fig 3B). Linear regression analysis (Fig 3C) showed a significant negative correlation (R^2^ = 0.91, p = 0.045) between cluster size of frontal cortex activation and amount of loudness maintenance that occurred at 35–38 sec of MTC stimulation taken from Fig 1. We presented the MTC stimulus 75 sec before PET acquisition to ensure that sufficient adaptation had occurred that could then be detected in the PET scans. Since we did not initially know which time point or time range of loudness adaptation shown in Fig 1 corresponds to the cortical activation pattern observed during the 90-sec PET acquisition, we analysed all possible time ranges and identified the one (i.e., 35–38 sec) that exhibited the strongest negative correlation as shown in Fig 3C. At that time point (35–38 sec) substantial loudness adaptation—if present—was already reached as can be seen in Fig 1. Fig 3D demonstrates that selecting a time point even out to 180 sec from Fig 1, which was the maximum time point we measured for loudness adaptation, we still observed a similar but non-significant trend (R^2^ = 0.80, p = 0.106) between cluster size of frontal cortex activation and amount of loudness maintenance.

Unlike speech stimuli, MTC stimulation elicited almost exclusively unilateral activation of the temporal cortex (see Table 2). Patient 1, 2, and 3 are implanted in the left cochlea or brainstem and caused MTC activation of the right temporal cortex, consistent with excitatory pathways crossing over to the contralateral side above the brainstem level. Patient 5 is implanted in the left midbrain and actually exhibited deactivation of the left temporal cortex, which may reflect ipsilateral stimulation effects of a region beyond the decussating pathways. What is particularly interesting is that stimulation of the same set of electrode sites within an implanted region in each patient with speech stimuli rather than the MTC somehow elicited bilateral activation of the temporal cortices (compare Table 1 and Table 2), demonstrating the importance of the pattern of stimulation across neurons in effectively transmitting sound information to higher perceptual regions.

### Differences in activation between speech and multi-tone complex stimulation

Speech stimulation in three patients who exhibited loudness adaptation (Patient 1, 3 and 5) revealed bilateral auditory cortex activation (mainly in BA 21 and 22) when the data analysis was performed in comparison to the MTC stimulation condition instead of the silence condition (Table 4).

**Table 4:** Activation in auditory cortex during speech stimulation versus multi-tone complex stimulation.

Patient no., auditory implant, speech score	Activation in PET ^a^									
	Side	x ^b^	y ^b^	z ^b^	Z value	T value	BA41 ^c^	BA42 ^c^	BA22 ^c^	BA21 ^c^
No. 1, CI left, 82%	left	-70	-38	4	5.22	14.23	0	0	959	1414
	right	64	-10	0	5.75	20.01	0	0	846	1216
No. 2, ABI 1 left, 58%	left	-64	-50	12	4.14	9.28	0	59	365	394
	right	58	2	52	5.11	18.59	0	415	1358	1510
No. 3, ABI 2 left, 55%	left	-74	-30	16	6.62	30.30	0	0	991	1437
	right	34	42	-30	5.16	12.19	0	0	343	448
No. 5, AMI 2 left, 0%	left	-68	-54	-14	5.32	15.10	256	407	1145	2259
	right	64	-50	14	4.43	8.88	0	133	256	154

^a^ Threshold for statistical inferences: p<0.001.

^b^ MNI coordinates.

^c^ Voxels in Brodmann Areas.

This result corroborates that auditory cortex activity is reduced down to or even lower than baseline level during MTC stimulation when loudness adaptation has occurred. Moreover, MTC stimulation in comparison to speech stimulation was associated with frontal cortex activation (in BA 9 and/or 10) in patients with loudness adaptation (which was not present in Patient 2 without loudness adaptation; Table 5), suggesting that during loudness adaptation this frontal cortical region may have a specific down-regulatory impact on the auditory cortex.

**Table 5:** Activation in frontal cortex (BA 9, 10) during multi-tone complex stimulation versus speech stimulation.

Patient no., auditory implant, adaptation	Activation in PET ^a^							
	Side	x ^b^	y ^b^	z ^b^	Z value	T value	BA9 ^c^	BA10 ^c^
No. 1, CI left, partial	left						0	0
	right	36	50	40	5.16	13.69	1239	0
No. 2, ABI 1 left, none	left						0	0
	right						0	0
No. 3, ABI 2 left, partial	left	-24	72	8	4.31	7.72	0	345
	right						0	0
No. 5, AMI 2 left, complete	left	-2	22	-38	5.07	13.00	114	426
	right						0	0

^a^ Threshold for statistical inferences: p<0.001.

^b^ MNI coordinates.

^c^ Voxels in Brodmann Areas.

## Discussion

In the present study, correlates in central processing of auditory stimuli, specifically MTC and speech stimuli, presented through an auditory implant were investigated using PET. The objective was to detect diagnostic patterns for function (i.e., speech understanding) and dysfunction (i.e., loudness adaptation) in individual patients and across implant types, including the CI, ABI, and AMI.

### PET imaging of speech perception

Consistent with previous studies [4, 6, 8, 11, 12, 30, 31], we observed a correlation of greater bilateral activation of auditory cortex and association areas, including the TVA, with greater speech perception performance across implant types. Our findings also reveal the importance of activity within the primary auditory cortex (i.e., BA 41 and 42) for speech understanding, since increased activation of this specific region was more strongly correlated with speech score compared to regions that included BA 21 and 22. Moreover, we were able to present for the first time PET activation of temporal cortical regions in AMI patients in response to speech stimuli, in which low speech scores corresponded to weak or no auditory cortex activation.

### Cortical activation and mechanisms for processing relevant versus irrelevant auditory inputs

A unique aspect of our study was the ability to assess and compare activated cortical regions in response to speech stimuli versus a meaningless MTC stimulus that could induce loudness adaptation. This comparative protocol revealed five main findings. First, the MTC stimulus mainly elicited unilateral activation or deactivation of the auditory cortex (Table 2). This unilateral response corresponded to the expected side of activity based on the decussating projections through the ascending auditory pathways. Second, the patients that experienced greater loudness adaptation exhibited weaker auditory cortex activation and even deactivation relating to that unilateral response (Table 2). Third, greater adaptation (i.e., less loudness maintenance) also corresponded to greater activation of the ventral frontal cortex (Table 3 and Fig 3). There appears to be greater activity within the frontal cortex that is ipsilateral to the unilateral response in the auditory cortex. Fourth, speech stimuli elicited bilateral activation of the auditory cortex, except for patient 5 who had no detectable auditory cortical response and no speech understanding (Table 1). Considering that each patient was stimulated with the same electrode sites but just with a different inputted sound stimulus (i.e., speech versus MTC stimulation) demonstrates that the type of electrical pattern presented across neurons can lead to bilateral or unilateral activity in the auditory cortex. If the stimulus is not appropriately activating a given population of auditory neurons, it may lead to only unilateral activity or even a shutdown of response in the auditory cortex. Even though Patient 5 was presented with speech stimuli, it may not have appropriately activated the ascending pathway and/or activated the appropriate neurons to maintain an auditory cortical response. Fifth, bilateral auditory cortex activation is not sufficient for enabling speech perception. We observed greater bilateral auditory cortex activation for patients who achieved higher speech scores (Table 1 and Fig 2). However, we also observed that bilateral auditory cortex activation was still possible even though the patient achieved almost no speech understanding (Patient 4 in Table 1). Consistent with previous studies [4, 11–13], our data suggest that extended bilateral activation of auditory cortex and association areas, such as the TVA, are required for high levels of speech understanding.

One could consider in some patients and for some neural regions that continuous electrical stimuli transmitted through implant electrodes may not adequately mimic physiologic activation in downstream signalling. As a result, only unilateral activation or even deactivation can occur within the auditory cortex. This lack of cortical activation is not solely due to stimulating the wrong neurons since activating those neurons surrounding the same electrodes but just with different stimuli (e.g., speech versus MTC stimulation) led to drastically different cortical responses, demonstrating the importance of the spatial and temporal pattern of activity across neurons for transmitting sound information to higher perceptual regions. At least for speech stimuli, if there is sufficient activation of the appropriate neurons, then bilateral activation occurs within the auditory cortex spanning across the TVA, which appears to be necessary for good speech understanding. If there is insufficient or inappropriate activation of the ascending pathway, such as caused by irrelevant stimuli (e.g., our MTC stimulus) or even pathogenic patterns (e.g., associated with tinnitus or hyperacusis), the ventral prefrontal cortex could be involved with gating or suppressing that activity, and thus reduce or shift attention away from its perceptual effect. The ventral prefrontal cortex may also be involved with limiting auditory cortex activation to only a unilateral response for the meaningless MTC stimulus. However, the fact that Patient 2 had no loudness adaptation or no frontal cortex activation yet still only had unilateral auditory cortex activation (see Tables 2 and 3) suggests the unilateral effect is not solely caused by a prefrontal cortical mechanism.

There are several studies that support the proposed role of the prefrontal cortex in modulating or gating the auditory cortex. Lesions within frontal cortical regions have shown increased auditory cortex activation, suggesting the role of the frontal cortex in suppressing or gating auditory cortex activity [22, 23]. Electrophysiological and behavioural studies in non-human primates provide evidence that the prefrontal cortex, including BA 10, plays a role in the memory-based analysis of auditory objects [32]. Further evidence supporting the role of the frontal cortex in perceptive mechanisms comes from electrophysiological measurements in humans for the so-called mismatch negativity response. This is a phenomenon observed in association with an auditory stimulus not fitting to the prior context. fMRI provides evidence that it originates partly from the frontal cortex [33]. Additional evidence that the frontal cortex is involved in auditory cortex modulation is provided by a PET study from Morris et al. [34]. They measured regional brain activity in volunteers during classical conditioning with a high frequency (8000 Hz) or low frequency (200 Hz) tone at an intensity level of 70 dB with an aversive 100 dB white noise burst, and they observed conditioning related modulation of auditory cortex responses. The modulated region of the auditory cortex co-varied with activity in the basal forebrain and the orbitofrontal cortex. Therefore, the frontal activation observed in our study in the situation of loudness adaptation during MTC perception might reflect a frontal up-regulation to actively shut down auditory cortex activity due to a non-interpretable signal into the auditory network caused by artificial implant stimulation. It is also possible that this frontal up-regulation is due to deafferentation from adequate network input of the auditory cortex or even increased attention-related frontal activity associated with a vanishing signal within the auditory cortex.

## Conclusions

The present study demonstrates that PET imaging can reveal in individual users of auditory implants specific patterns of cerebral cortex activity corresponding to speech understanding as well as adapting or inappropriate auditory inputs. Therefore, PET has potential as a clinical tool for understanding and identifying appropriate neural targets and stimulation strategies for auditory implant rehabilitation. PET also has the potential for guiding stimulation approaches for treating tinnitus. One intriguing finding was the increase in ventral frontal cortex activity with a decrease in auditory cortex activity during loudness attenuation of an irrelevant stimulus. Tinnitus has been linked to hyperactivity and abnormal patterns within the ascending pathways up to the auditory cortex [35–39]. Neural stimulation approaches could activate the frontal cortex, which in turn may suppress and/or alter the abnormal patterns within the auditory cortex associated with the tinnitus percept [40, 41]. In a broader sense and considering the increasing number of individuals receiving deep brain and cortical implants for various conditions [42], there will be an even larger clinical opportunity for using PET imaging beyond auditory applications that can overcome artefact and safety issues associated with fMRI approaches in neural implant patients.
